# A dataset of physico-chemical properties, extractable organic N, N mineralization and physical organic matter fractionation of soils developed on loess silts, crystalline rocks and sedimentary rocks

**DOI:** 10.1016/j.dib.2023.109776

**Published:** 2023-11-07

**Authors:** Thierry Morvan, Yvon Lambert, Philippe Germain, Blandine Lemercier, Mariana Moreira, Laure Beff

**Affiliations:** a SAS, INRAE, Institut Agro, 35000 Rennes, France; b Chambres d'Agriculture de Bretagne, 35000 Rennes, France; c SAS, Institut Agro, INRAE, 35000 Rennes, France

**Keywords:** Physico-chemical soil properties, Soil microbial biomass, N mineralization incubation, Physical organic matter fractionation, Extractable organic N

## Abstract

A network of 137 cultivated fields covering the wide diversity of soils, crop rotations and cropping practices throughout the region of Brittany (France) was monitored to collect data on soil organic nitrogen (SON) mineralization and to identify the factors that explain the observed variability. The dataset presented in this article contains all of the information about the soils, which were subjected to pedological description and in-depth analysis of their topsoil properties. The topsoil (0–30 cm) was sampled by mixing 30 samples to obtain one composite per field, which was divided into one sub-sample sieved at 5 mm to analyze soil microbial biomass (SMB) and SON mineralization via anaerobic incubation, and one subsample dried at 40 °C and sieved at 2 mm. The physico-chemical analyses included the particle-size distribution of five fractions; organic matter (OM); organic C; organic N; pH (water); pH KCl; CEC (Metson); CEC (hexamminecobalt); exchangeable Al, Ca, Fe, K, Mg, Mn and Na (hexamminecobalt); Olsen P; Dyer P; and total Al, Ca, Fe, K, Mg, Mn, Na and P. Physical OM fractionation was used to characterize the 200–2000 µm and 50–200 µm fractions of particulate organic matter (POM). Finally, three chemical methods were used to determine extractable organic nitrogen (EON): hot KCl, hot water and phosphate buffer tests. This dataset covers a wide range of pedological situations and cropping systems, and is of great interest to scientists searching for soil properties that can explain SON mineralization. It provides original data on EON indices, SMB and multiple forms of P. This paper supports and supplements information presented in a previous article [1].

Specifications Table

**Table:** 

Subject	Agricultural Sciences
Specific subject area	Soil Science
Data format	Table, Figure, Image
Type of data	Raw, Analyzed
Data collection	Field sampling of a network of 137 cultivated fields covering a wide diversity of soils and cropping history, followed by laboratory analysis
Data source location	The data were collected from a network monitored throughout the region of Brittany (France) by the Regional Chamber of Agriculture and INRAE. The dataset provides GPS coordinates of the experimental fields.
Data accessibility	This paper provides the analyzed data. The raw data have been deposited in a public repository. Repository name: Data INRAE Data identification number: 10.57745/DGIPGR Direct URL to data: https://doi.org/10.57745/DGIPGR
Related research article	T. Morvan, L. Beff, Y. Lambert, B. Mary, P. Germain, B. Louis, N. Beaudoin, 2022. An original experimental design to quantify and model net mineralization of organic nitrogen in the field. Nitrogen, 3, 197–212. https://doi.org/10.3390/nitrogen3020015

## 1 Value of the Data

• This dataset of physico-chemical properties, SMB, SON mineralization and EON is of great interest given the wide diversity of soil types and the cropping history of the fields. It also provides original data on multiple forms of P.
• The dataset would be useful to agronomists and soil scientists who investigate the soil parameters that drive SON mineralization. These data also enable other researchers to develop data-analysis approaches that differ from that presented in the related article.
• This dataset could be valuable in future meta-analyses that seek to understand the influence of soil parent material and soil type on soil physico-chemical properties, SMB and SON mineralization.
• This comprehensive dataset of physical, chemical and biological properties of soils is combined with accurate reference values for soil mineralization, measured over three consecutive years.
• This dataset provides complete information about soil properties, only some of which were presented and used in a previous article [1], and supplements the data on N balances presented in another data paper [2].

## 2 Data Description

**Fig. 1:**
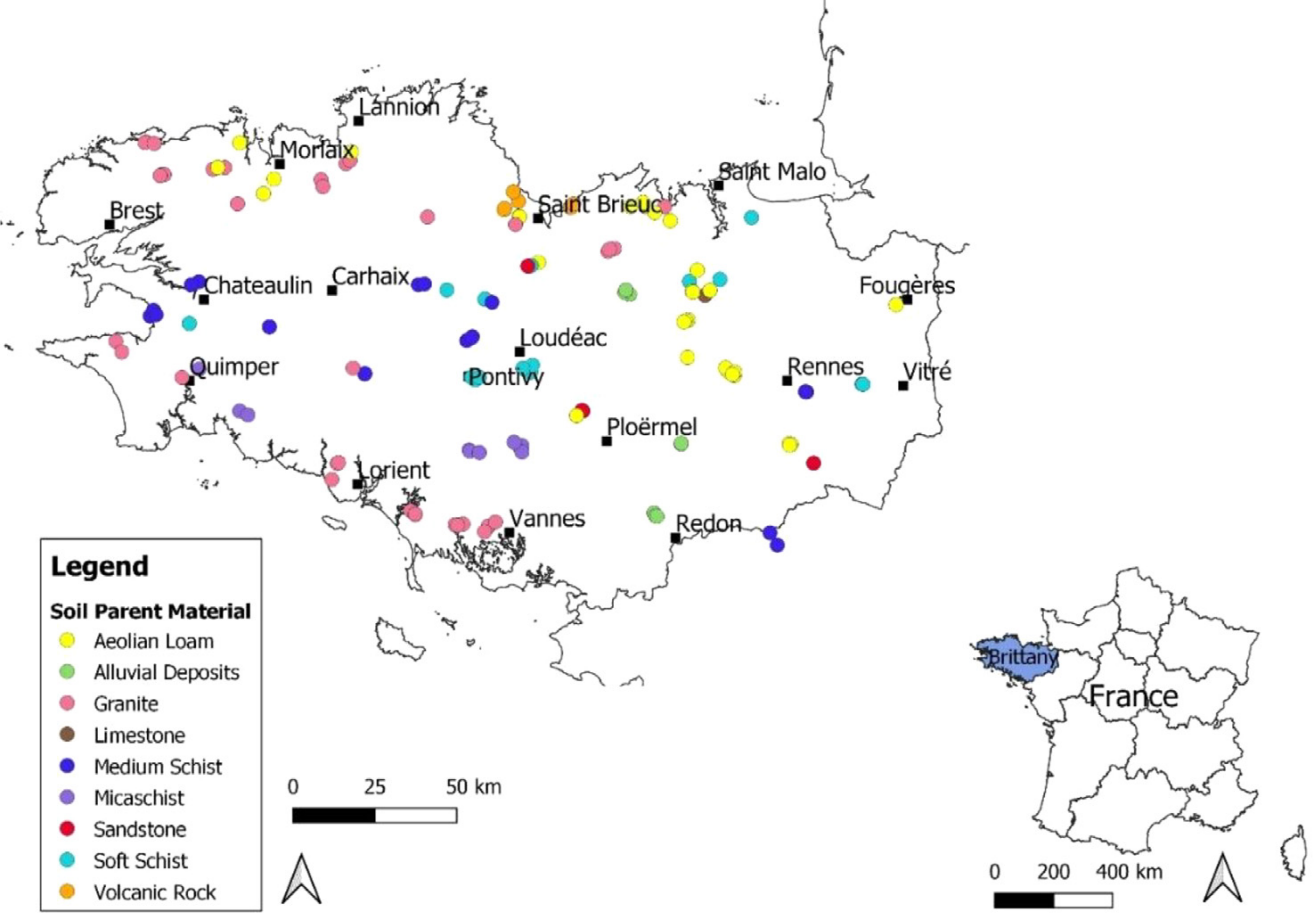
Locations and soil parent materials of the network fields.

**Fig. 2:**
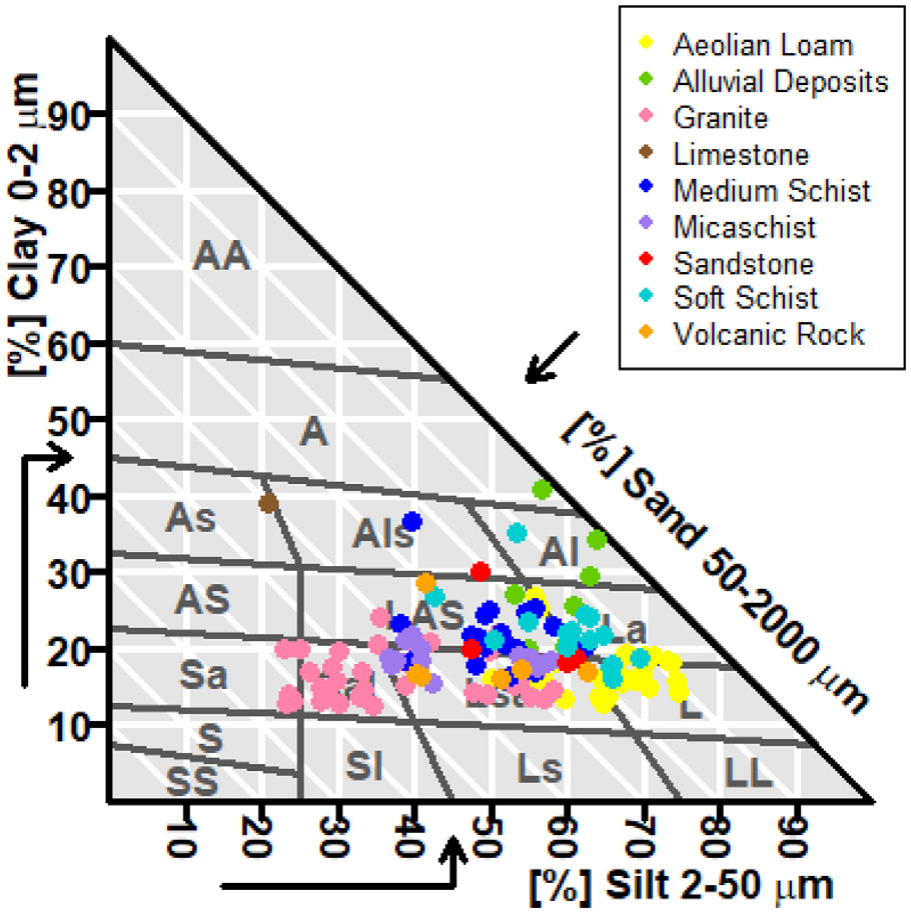
Textures (according to the GEPPA texture triangle) of the topsoil (0–30 cm) and the soil parent materials of the network fields.

**Fig. 3:**
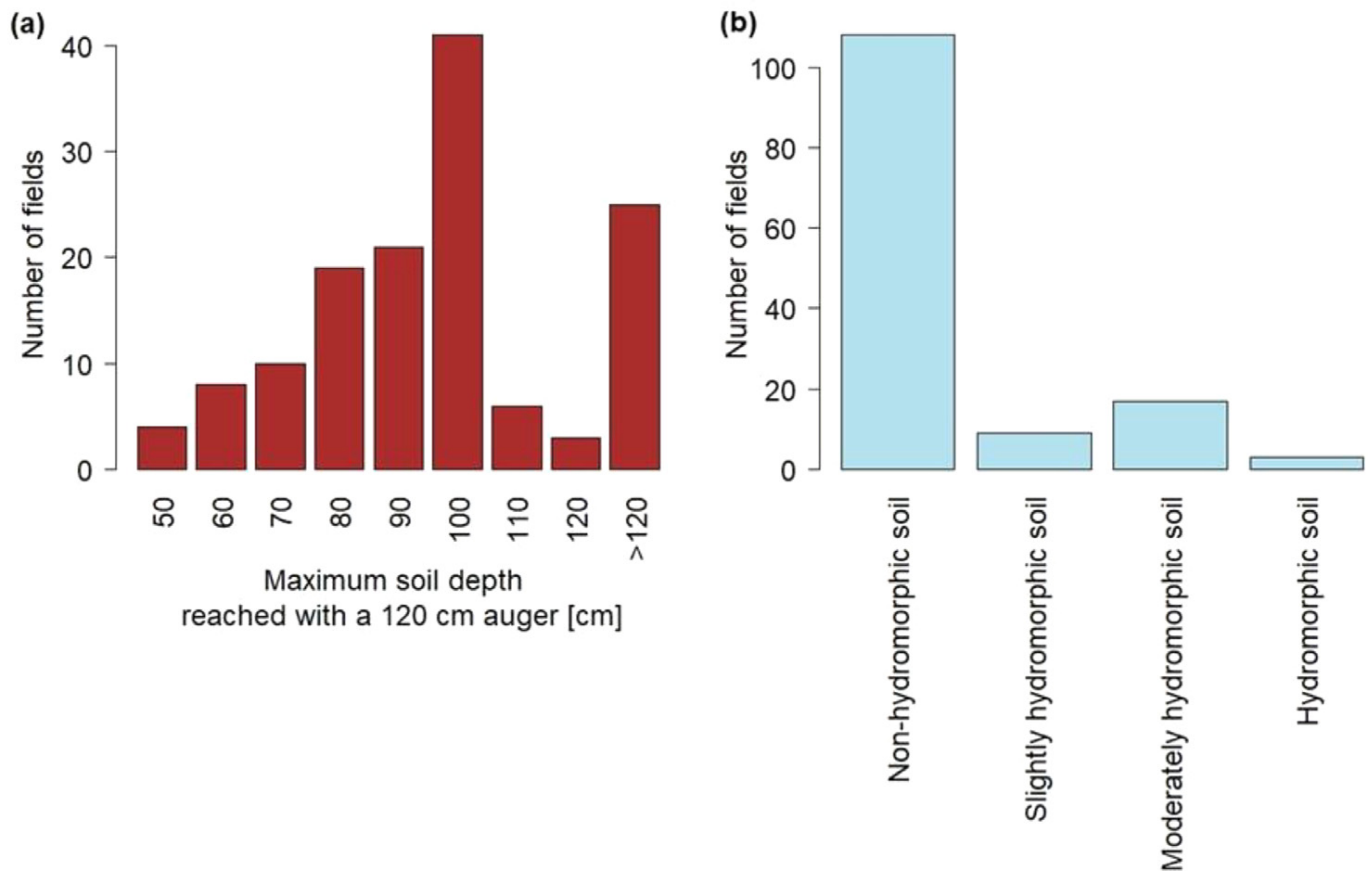
Number of soils (a) of a given maximum depth and (b) in each hydromorphic class.

**Fig. 4:**
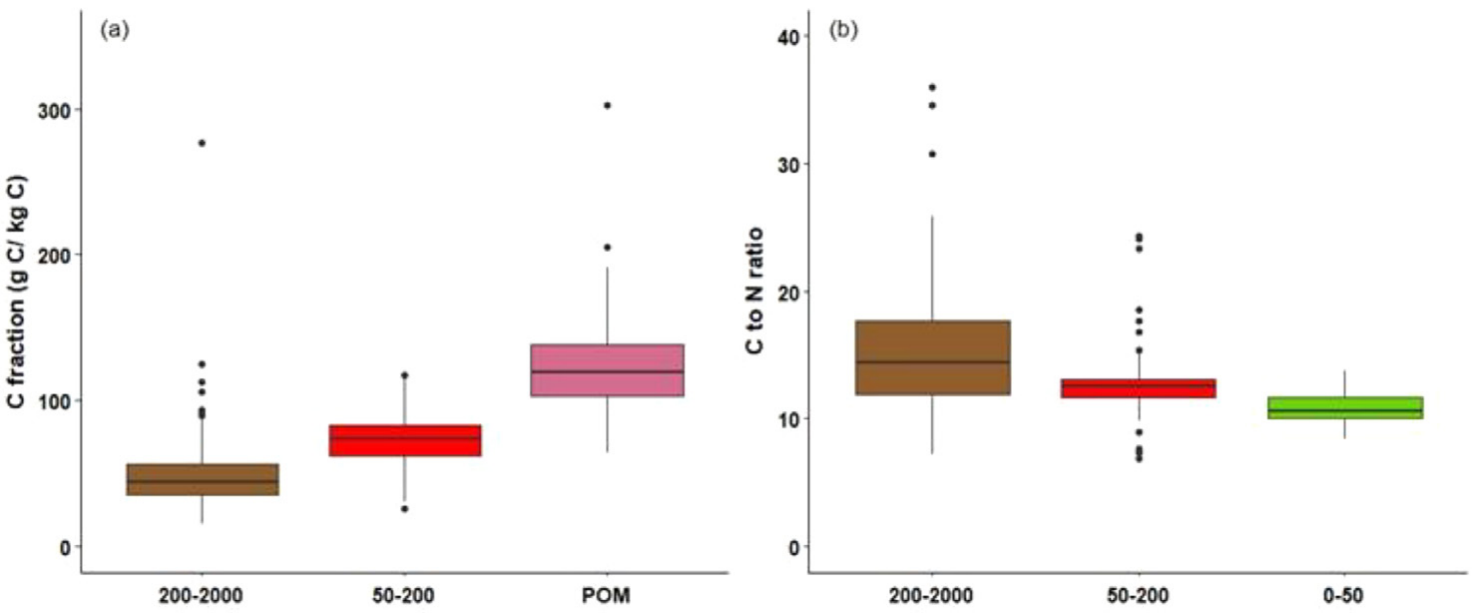
Boxplots of the (a) C 200–2000 µm, 50–200 µm and POM fractions and (b) C to N ratio of the 200–2000 µm, 50–200 µm and 0–50 µm fractions. Whiskers represent 1.5 times the interquartile range.

**Fig. 5:**
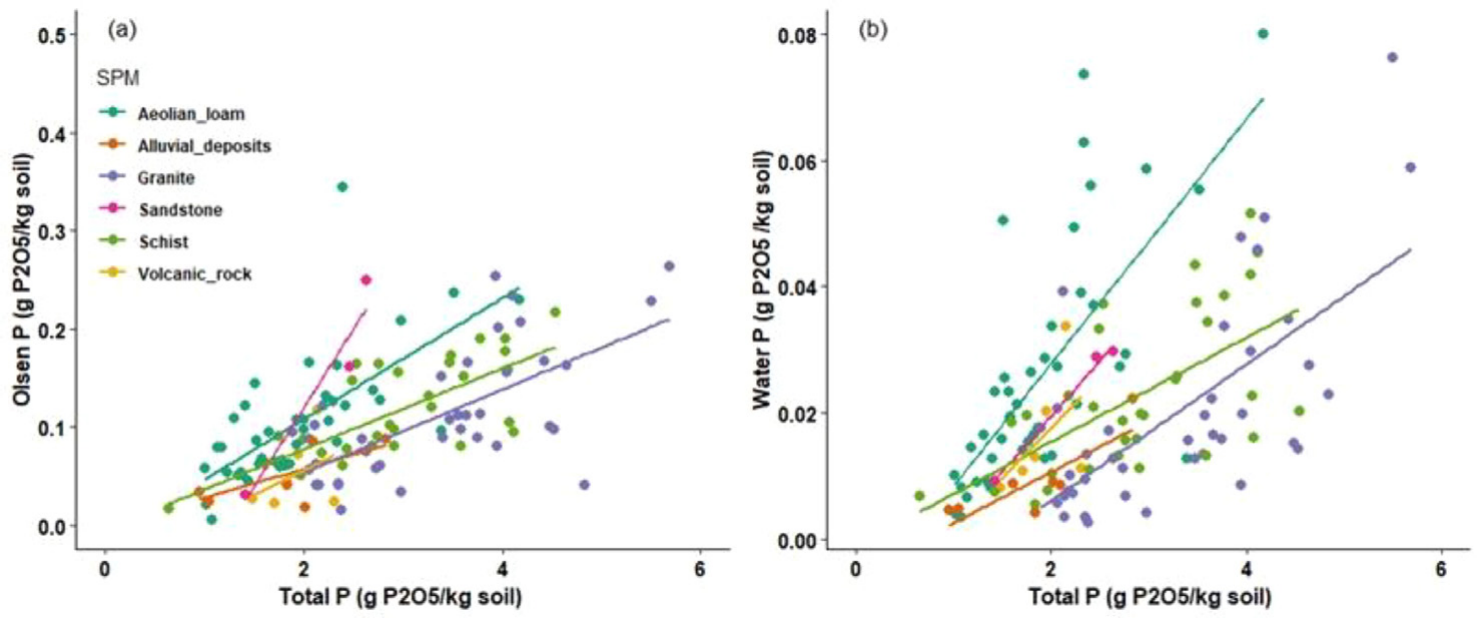
Relation between (a) total P and Olsen P, and (b) total P and water P by soil parent material (lines indicate linear regressions.

This article includes raw data, tables and figures that describe properties of the soils in the network, including the locations and soil parent materials (SPM) of the network fields (Fig. 1); the distribution of topsoil (0–30 cm) textures as a function of SPM (Fig. 2); the distribution of soil depth and hydromorphic characteristics (Fig. 3); summary statistics of physico-chemical properties of the soils (Table 1), SMB, N mineralization during incubation and EON (Table 2) and organic matter physical fractions (Table 3). Table 4 shows correlations between N mineralization during incubation, organic C, total N and EON. Fig. 4 shows the distribution of particulate organic matter fractions and their C:N ratio, and Fig. 5 shows relationships between total P, Olsen P and water P.

**Table 1:** Summary statistics of the physico-chemical properties of the topsoil (0–30 cm): particle-size distribution, chemical element contents (g kg^−1^ soil), Metson and hexamminecobalt cation exchange capacity (CEC, meq 100 g^-1^ soil), and pH. SD = standard deviation.

Property	Mean	SD	Min.	Max.
Clay	193.8	51.8	124.0	408.0
Fine silt	247.8	87.5	98.0	512.0
Coarse silt	268.3	118.8	66.0	534.0
Fine sand	125.6	53.6	4.0	273.0
Coarse sand	164.5	129.3	7.0	517.0
pH	6.1	0.5	4.8	7.9
pH KCl	5.1	0.6	3.9	7.5
CEC Metson	9.8	2.5	5.5	17.2
CEC hexamminecobalt	6.3	2.6	2.8	20.0
Organic matter	33.9	11.3	15.3	78.9
Organic C	19.8	6.5	8.8	45.6
Total N	1.81	0.57	0.87	3.86
Olsen P	0.104	0.063	0.005	0.345
Dyer P	0.366	0.248	0.031	1.170
Total P	2.582	1.053	0.648	5.690
Water P	0.022	0.016	0.003	0.080
Total Al	58.61	14.18	35.60	94.00
Total Ca	4.49	4.63	1.46	31.90
Total Fe	27.28	11.93	8.23	73.10
Total K	17.37	4.08	7.77	33.00
Total Mg	4.53	2.75	1.46	14.70
Total Mn	5.93	3.62	0.84	20.80
Total Na	7.91	3.47	2.14	18.00
Exchangeable Al	0.154	0.191	0.020	1.430
Exchangeable Ca	5.879	2.341	2.030	15.000
Exchangeable Fe	0.009	0.005	0.005	0.031
Exchangeable Mg	0.666	0.451	0.232	4.700
Exchangeable Mn	0.089	0.070	0.006	0.388
Exchangeable K	0.329	0.169	0.069	1.270
Exchangeable Na	0.063	0.039	0.026	0.341

**Table 2:** Summary statistics of the soil microbial biomass (SMB), N mineralization during incubation and extractable organic N. SD = standard deviation.

Item	Unit	Mean	SD	Min.	Max.
SMB	mg C kg^−1^	169.7	45.4	81.6	405.0
N incubation	mg N kg^−1^	22.1	6.7	8.7	66.0
Hot KCl	mg N kg^−1^	22.6	6.6	10.9	49.5
Hydr KCl	mg N kg^−1^	15.1	5.2	2.2	39.0
Phosphate buffer	mg N kg^−1^	26.8	8.1	7.9	57.8
Hot-water N	mg N kg^−1^	75.0	20.1	40.0	155.0
Hot-water C	mg C kg^−1^	770.9	245.9	343.0	1846.5

**Table 3:** Summary statistics of the organic matter fractions by element.

Element	Particle size	Unit	Mean	SD	Min.	Max.
Carbon	200–2000 µm	g C kg^−1^C	50.9	28.0	15.1	277.1
	50–200 µm	g C kg^−1^C	73.0	19.6	25.6	117.6
	0–50 µm	g C kg^−1^C	876.1	34.8	608.4	927.2
	POM	g C kg^−1^C	123.9	34.8	72.8	391.6
Nitrogen	200–2000 µm	g N kg^−1^ N	37.5	17.7	11.2	127.0
	50–200 µm	g N kg^−1^ N	64.5	18.6	17.9	111.2
	0–50 µm	g N kg^−1^ N	898.0	21.3	810.7	936.6
	POM	g N kg^−1^ N	102.0	21.3	63.4	189.3

**Table 4:** Correlations between N mineralization during incubation, organic C, total N and extractable organic N (EON) (Hot_KCl, Hydr_KCl, Phosphate_buffer, Hot_water_N, Hot_water_C). SMB = soil microbial biomass.

	N incubation	SMB	Organic_C	Total_N	Hot_KCl	Hydr_KCl	Phoshate buffer	Hot_water N	Hot_water C
N incubation	1								
SMB	0.54	1							
Organic_C	0.52	0.37	1						
Total_N	0.56	0.49	0.93	1					
Hot_KCl	0.60	0.43	0.84	0.87	1				
Hydr_KCl	0.61	0.43	0.84	0.86	0.91	1			
Phoshate_buffer	0.47	0.46	0.72	0.77	0.80	0.74	1		
Hot_water_N	0.54	0.59	0.59	0.68	0.66	0.63	0.59	1	
Hot_water_C	0.45	0.56	0.56	0.65	0.61	0.57	0.63	0.81	1

The dataset consists of four .csv files that contain raw data: Soil_description.csv (Table 5), Physico_chemical_properties.csv (Table 6), EON_physical_fractionation.csv and Bulk_density.csv (Table 7), a file that contains the metadata and a .zip file that contains photographs of the soil profiles*.* The dataset is available via the Data INRAE portal.

**Table 5:** Contents of the Soil_description.csv file of the dataset.

File name	Variable name	Content
**Soil_description.csv**	Id_field	Field identifier
	SPM	Soil parent material
	Hydromorphy	Degree of soil hydromorphy
	Solum	Type of soil profile development
	Depth	Soil depth (cm)
	RAW	Estimated readily available water content (mm)
	Limits_layer1	Upper and lower depth of soil layer 1
	Texture_layer1	Texture of soil layer 1
	Hydromorphy_layer1	Hydromorphy in soil layer 1
	Stoniness_layer1	Stones in soil layer 1
	Limits_layer2	Upper and lower depth of soil layer 2
	Texture_layer2	Texture of soil layer 2
	Hydromorphy_layer2	Hydromorphy in soil layer 2
	Stoniness_layer2	Stones in soil layer 2
	Limits_layer3	Upper and lower depth of soil layer 3
	Texture_layer3	Texture of soil layer 3
	Hydromorphy_layer3	Hydromorphy in soil layer 3
	Stoniness_layer3	Stones in soil layer 3
	Limits_layer4	Upper and lower depth of soil layer 4
	Texture_layer4	Texture of soil layer 4
	Hydromorphy_layer4	Hydromorphy in soil layer 4
	Stoniness_layer4	Stones in soil layer 4

**Table 6:** Contents of the Physico_chemical_properties.csv file of the dataset.

File name	Variable name	Content
**Physico_chemical_properties.csv**	ID_field	Field identifier
	X_WGS84	X WGS84 coordinate
	Y_WGS84	Y WGS84 coordinate
	SPM	Soil parent material
	Clay	Clay (g kg^−1^)
	Fine_Silt	Fine_Silt (g kg^−1^)
	Coarse_Silt	Coarse_Silt (g kg^−1^)
	Fine_Sand	Fine_Sand (g kg^−1^)
	Coarse_Sand	Coarse_Sand (g kg^−1^)
	pH_water	Water pH
	pH_KCl	KCl pH
	CEC_Metson	Metson CEC (meq 100 g^−1^ soil)
	CEC_hexamminecobalt	Hexamminecobalt CEC (meq 100 g^−1^ soil)
	Organic_matter	Soil organic matter (g kg^−1^)
	Organic_C	Soil organic C (g kg^−1^)
	Total_N	Soil N content (g kg^−1^)
	P_Olsen	Olsen P (g P_2_O_5_ kg^−1^)
	P_Dyer	Dyer P (g P_2_O_5_ kg^−1^)
	Total_P2O5	Total P (g P_2_O_5_ kg^−1^)
	P2O5_water	Water P (g P_2_O_5_ kg^−1^)
	Total_Al	Total Al (g kg^−1^)
	Total_Ca	Total Ca (g kg^−1^)
	Total_Fe	Total Fe (g kg^−1^)
	Total_K	Total K (g kg^−1^)
	Total_Mg	Total Mg (g kg^−1^)
	Total_Mn	Total Mn (g kg^−1^)
	Total_Na	Total Na (g kg^−1^)
	Exchangeable Al	Hexaminnecobalt exchangeable Al (g kg^−1^)
	Exchangeable Ca	Hexaminnecobalt exchangeable Ca (g kg^−1^)
	Exchangeable Fe	Hexaminnecobalt exchangeable Fe (g kg^−1^)
	Exchangeable Mg	Hexaminnecobalt exchangeable Mg (g kg^−1^)
	Exchangeable Mn	Hexaminnecobalt exchangeable Mn (g kg^−1^)
	Exchangeable K	Hexaminnecobalt exchangeable K (g kg^−1^)
	Exchangeable Na	Hexaminnecobalt exchangeable Na (g kg^−1^)

**Table 7:** Contents of the EON_physical_fractionation.csv and Bulk_density.csv files of the dataset.

File name	Variable name	Content
**EON_physical_fractionation.csv**	SMB	Soil microbial biomass (mg C kg^−1^)
	N_incubation	N incubation (mg N kg^−1^)
	Hot_KCl	Hot KCl extractable N (mg N kg^−1^)
	Hydr_KCl	Hot KCl hydrolyzable N (mg N kg^−1^)
	Phosphate_buffer	Phosphate-borate distillable N (mg N kg^−1^)
	Hot_water_N	Hot-water extractable N (mg N kg^−1^)
	Hot_water_C	Hot-water extractable C (mg C kg^−1^)
	C 200–2000	C 200–2000 µm (g C kg^−1^C)
	C 50–200	C 50–200 µm (g C kg^−1^C)
	C 0–50	C 0–50 µm (g C kg^−1^C)
	N 200–2000	N 200–2000 µm (g N kg-1 N)
	N 50–200	N 50–200 µm (g N kg^−1^ N)
	N 0–50	N 0–50 µm (g N kg^−1^ N)
	POM-C	Particulate organic matter C (g C kg^−1^C)
	POM-N	Particulate organic matter N (g N kg^−1^ N)
**Bulk_density.csv**	BD_Layer_1	Bulk density layer 0–30 cm (g cm^3–1^)
	BD_fine_Layer_1	Bulk density of fine soil layer 0–30 cm (g cm^3–1^)
	BD_Layer_2	Bulk density layer 30–60 cm (g cm^3–1^)
	BD_fine_Layer_2	Bulk density of fine soil layer 30–60 cm (g cm^3–1^)
	BD_Layer_3	Bulk density layer 60–90 cm (g cm^3–1^)
	BD_fine_Layer_3	Bulk density of fine soil layer 60–90 cm (g cm^3–1^)

## 3 Experimental Design, Materials and Methods

### 3.1 Network description and soil characterization

A network of 137 fields was established in Brittany to quantify SON mineralization during three consecutive years. To this end, the fields were cropped with silage maize without any mineral or organic fertilization, and SON mineralization was calculated from the end of winter to the beginning of autumn from the mineral N mass balance of the maize crop. The fields were chosen in order to study two main factors that determine N mineralization – soil properties (physical, chemical and biological) and the cropping system – with the objective of obtaining a number of fields representative of the variability of soils and cropping systems in the region. The sampling based on these two factors led to the selection of 37 soils developed on parent material consisting of schist (21 medium and 16 soft), 36 on granite, 32 on aeolian loam (23 deep and 19 moderately deep), 13 on micaschist, 6 on volcanic rock, 4 on sandstone, 1 on limestone and 8 on alluvium (i.e. valley bottoms) (Figs. 1 and 2), which corresponds closely to the distribution of soil parent materials in Brittany soils [3]. This network also enabled us to assess the diversity of rotations and organic fertilization practices in the region's cropping systems [2].

The plots were agricultural fields used to grow maize; thus, all had moderately deep to deep soil, and most of the soils were non-hydromorphic or slightly hydromorphic (Fig. 3b).

Soils were described from auger-extracted profiles. To ensure that the network's soil was described as consistently as possible, the same soil scientist (YL) produced and described all soil profiles. Each soil was described in general terms: parent material, degree of hydromorphy, type of solum and depth [4]. The auger was able to extract soil profiles down to a depth of 120 cm. All soils deeper than 120 cm were thus noted (Fig. 3a).

More specifically, soil layers were characterized by their thickness, presence of stones, hydromorphy and texture. In addition, the readily available water content (i.e. soil water that plants can extract easily) was estimated from its thickness and texture of the whole soil profile [4].

Bulk density and fine-earth bulk density were determined as a proportion of the total and fine-earth mass (both dried at 105 °C), respectively, and sample volumes were measured using the cylinder method (1237 cm^3^) following standard NF EN ISO 11272 [5].

### 3.2 Topsoil sampling

The experiment was conducted on an area of 1485 m^2^ (33 m × 45 m) precisely georeferenced within the fields, divided into three subplots of 45 m^2^ (6.0 m × 7.5 m) in the middle for triplicate measurements. The topsoil (0–30 cm) was sampled by removing 10 cores along a transect with a 3 cm diameter auger on each of the 45 m^2^ subplots. The 30 soil samples were then mixed to obtain one composite, which was stored at 4 °C for no more than 3 days. The samples were then divided by successive quartering, with one half sieved at 5 mm and sent to laboratories for biological analysis. The other half was dried at 40 °C and sieved at 2 mm in perforated titanium drums fitted with cylindrical titanium rollers, and then a 500 ml subsample of the homogenized fine soil was taken for physical and chemical analysis. An additional 30 g sample was taken and ground to a particle size of less than 250 µm in a planetary mill. The soil sieved to 2 mm was analyzed when the sample mass was > 1.5 g, while the soil ground to 250 µm was analyzed when the sample mass was < 1.5 g.

### 3.3 Physico-chemical analysis

Soil texture was analyzed by measuring the particle size of five fractions: clay (< 2 µm), fine silt (2–20 µm), coarse silt (20–50 µm), fine sand (50–200 µm) and coarse sand (200–2000 µm), according to the standard NF X 31–107 [6].

SOM content was determined by grinding 1 g soil samples to 250 µm and carbonizing them at 550 °C. Organic C and total N contents of the soils were determined by grinding 1 g samples to 250 µm, followed by dry combustion, according to standards NF ISO 10694 [7] and NF ISO 13878 [8], respectively.

The cation exchange capacity (CEC) was determined by grinding 2.5 g soil samples to 2 mm and applying the Metson method, according to standard AFNOR NF X 31-130 [9]. The effective CEC of the soil was represented by the CEC (hexamminecobalt), which was measured after shaking 2.5 g soil samples in a hexamminecolbalt(III) chloride solution (50 mmol+ l-1), according to standard NF X 31-130 [9]. Exchangeable hexamminecolbalt ions Al, Ca, Fe, K, Mn and Na were quantified by inductively coupled plasma optical emission spectrometry and plasma microwave atomic emission spectroscopy.

Soil pH (water) was measured after air-drying 10 g samples, sieving them to 2 mm and suspending them in water in a 1:5 ratio (v/v), according to standard NF EN ISO 10390 [10]. Soil pH KCl was measured after air-drying 10 g samples, sieving them to 2 mm and suspending them in a 1 M KCl solution in a 1:5 ratio (v/v), according to standard NF EN ISO 10390 [10].

Assimilable P was determined by the Olsen method using 2.5 g soil samples, according to standard NF ISO 11263 [11], and by the Dyer method using 10 g soil samples, according to standard NF X 31–160 [12].

Total Al, Ca, Fe, K, Mg, Mn, Na and P were determined using inductively coupled plasma atomic emission spectroscopy after grinding 250 g soil samples to 250 µm and digesting them in hydrofluoric acid, according to standards NF X 31-147 [13] and NF ISO 14869-1 [14].

### 3.4 Biological measurements

SMB was determined using the chloroform fumigation extraction method [15] according to standard NF EN ISO 14240-2 [16]. Measurement of N mineralization under controlled laboratory conditions (N incubation) was based on adapting methods of [17,18]. First 20 g of fresh soil was incubated under anaerobic conditions, underwater, at 40 °C for 7 days. Ammonia N was then extracted in a KCl solution whose molarity was adjusted to obtain a final concentration of 1 M and a soil:solution ratio of 1:5. Any ammonia N present at the beginning of the incubation was extracted in the same manner. The amount of ammonia N was determined using colorimetry. The amount of N mineralized was determined as the amount of ammonia N after 7 days of incubation minus the initial amount of ammonia N in the sample.

## 4 Extractable Organic N Analysis

### 4.1 Hot-water-extractable C and N

Extraction with hot water solutions was used to measure extractable C (Hot_water_C) and extractable N (Hot_water_N) based on the procedure of [19]. A 7 g sample of dry soil sieved at 2 mm was suspended in 35 ml of distilled water in closed 40 ml vials. The vials were then incubated at 100 °C for 1 h on heating ramps. After cooling, the vials were centrifuged, and the organic C concentration of the supernatant was determined using a total organic C analyzer, and the total N concentration was determined using colorimetry after mineralizing the organic N in the solution, according to standard ISO 1570.

### 4.2 Hot KCl extractable NH_4_-N and hydrolyzable NH_4_

Hot KCl extractable NH_4__—_N and hydrolyzable NH_4_ were determined using an extraction method developed by [20]. A 3 g sample of dry soil sieved at 2 mm was placed in 20 ml of a 2 M solution of KCl. The suspended soil in solution was incubated at 100 °C in a water bath for 4 h. After cooling, the suspension was filtered through a Whatman 42 filter, and the NH_4__—_N concentration (Hot_KCl_NH4_) was determined using colorimetry. At the same time, the initial quantity of NH_4__—_N in the sample was determined by shaking a 3 g soil sample in 20 ml of 2 M KCl for 30 min, filtering it through at Whatman 42 filter and then using colorimetry. The amount of hydrolyzable NH_4_ (Hyd_KCl_NH4_) was calculated as the initial amount of NH_4__—_N in the sample minus Hot_KCl_NH4_.

### 4.3 Phosphate-borate distillable N (phosphate buffer test)

The amount of hydrolyzed N was determined by extracting it in a buffer solution of phosphate-borate, a method also developed by [21]. A 4 g sample of dry soil sieved at 2 mm was placed in 40 ml of a buffer solution of phosphate-borate (pH 11.2). The suspended soil in solution was distilled directly for 5 min, until 40 ml of distillate was collected in a beaker that contained 5 ml of boric acid. The amount of NH_4__—_N in the distillate was then determined using back titration with 0.0025 M sulfuric acid. At the same time, the initial quantity of NH_4__—_N in the sample was determined by directly distilling 4 g of soil in 20 ml of 2 M KCl and 0.2 mg of magnesium oxide, followed by back titration of the distillate as before. The amount of hydrolyzed N was calculated as the initial amount of NH_4__—_N in the sample minus the amount of total N in the buffer solution of phosphate-borate.

### 4.4 Physical OM fractionation

Physical fractionation was performed using the procedure of [22], which quantifies the OM content in three fractions: 20–2000 µm (coarse sand), 50–200 µm (fine sand) and 0–50 µm (silt and clay). POM equals the 50–2000 µm fraction. First, a 50 g sample of dry soil sieved at 2 mm was suspended in a sodium hexametaphosphate solution (1 g/L) along with 5 mm glass beads, to break up soil aggregates. The suspended soil in solution was shaken at room temperature for 16 h. It was then carefully sieved at 200 µm under running water to push particles smaller than 200 µm through the sieve, leaving the 200–2000 µm fraction. The same operation was performed with a 50 µm sieve to obtain the 50–200 µm fraction. Wet samples of the two fractions were dried in an oven at 105 °C. C and N contents of the samples were determined by grinding the samples finely, followed by dry combustion, according to standards NF ISO 10694 and NF ISO 13878. The proportions of C and N in each fraction was calculated from the mass of the fraction, its C and N contents and the initial C and N contents of the soil.

## Limitations

None.

## Ethics Statement

The authors have read and followed the ethical requirements for publication in Data in Brief and confirm that the current work does not involve human subjects, animal experiments, or any data collected from social media platforms.

## CRediT authorship contribution statement

**Thierry Morvan:** Conceptualization, Methodology, Validation, Formal analysis, Investigation, Data curation, Writing – original draft, Writing – review & editing, Visualization, Supervision, Project administration, Funding acquisition. **Yvon Lambert:** Methodology, Validation, Formal analysis, Investigation, Data curation, Supervision, Project administration, Funding acquisition. **Philippe Germain:** Investigation, Data curation. **Blandine Lemercier:** Validation, Formal analysis, Investigation, Data curation, Project administration, Funding acquisition. **Mariana Moreira:** Validation, Formal analysis, Investigation, Data curation. **Laure Beff:** Validation, Formal analysis, Investigation, Data curation, Writing – original draft, Visualization.

## Data Availability

A dataset of physico-chemical properties, extractable organic N, N mineralization and physical organic matter fractionation of soils developed on loess silts, crystalline rocks and sedimentary rocks (Original data) (data INRAE)
